# Correlation between the non-use of cooking oil fume extractors and bone mineral density in population aged 45 years and older in China: a cross-sectional study

**DOI:** 10.3389/fendo.2023.1280429

**Published:** 2024-01-04

**Authors:** Haitao Zhang, Binhao Shi, Chunchun Yuan, Chen Huang, Tingrui Huang, Zhangyu Liao, Wenhao Zhu, Wei Zhong, Hongbin Xu, Jiangxun Ji, Feihong Cai, Yue Chen, Pan Sun, Xianhui Zeng, Zhiwu Yang, Jing Wang, Bing Shu, Qianqian Liang, Qi Shi, Chuanglong Xu, Dezhi Tang, Yongjun Wang

**Affiliations:** ^1^ Longhua Hospital, Shanghai University of Traditional Chinese Medicine, Shanghai, China; ^2^ Spine Institute, Shanghai Academy of Traditional Chinese Medicine, Shanghai, China; ^3^ Shanghai University of Traditional Chinese Medicine, Shanghai, China; ^4^ Key Laboratory of Theory and Therapy of Muscles and Bones, Ministry of Education, Shanghai, China; ^5^ Ganzhou Nankang District Traditional Chinese Medicine Hospital, Ganzhou, China; ^6^ Shanghai Geriatric Institute of Chinese Medicine, Shanghai, China; ^7^ Ningxia Hospital of Traditional Chinese Medicine and Chinese Medicine Research Institute, Yinchuan, China

**Keywords:** bone mineral density, fume extractors, cross-sectional study, correlation, population

## Abstract

**Introduction:**

The correlation between the non-use of cooking oil fumes (COFs) extractors and bone mineral density (BMD) have not been clarified. Consequently, this study attempted to explore the impact of non-use COFs extractors on BMD in population aged 45 years and older based on a cross-sectional study.

**Methods:**

This study was a cross-sectional study within the framework of an ongoing prospective population-based cohort study in China. The multivariate linear regression models were used to evaluate the correlation between the non-use of fume extractors in family cooking and total lumbar spine (LS), femoral neck (FN), total hip BMD and levels of bone metabolism markers.

**Results:**

A total of 3433 participants were included in the final analyses, of which 2607 (75.93%) participants used fume extractors. The results of models indicated that there were significant correlations of the non-use of fume extractors on total LS BMD (β = -0.024, 95% CI, -0.036, -0.012, *p* < 0.001), PINP (β = 4.363, 95% CI, 2.371, 6.356, *p* < 0.001) and ALP (β = 4.555, 95% CI, 2.593, 6.517, *p* < 0.001) levels.

**Conclusions:**

This study verified that the use of fume extractors is an efficacious measure to prevent LS bone loss. For the sake of public bone health, people should install a fume extractor in the kitchen and use it routinely when cooking.

## Introduction

Osteoporosis (OP) is one of the common orthopedic metabolic diseases characterized by decreased bone mass, damage of bone tissue microstructure, and increased bone fragility (1, 2). OP has attracted widespread attention due to its high incidence and associated fracture risk in the middle-aged and elderly population. According to statistics, the incidence of OP and low bone mineral density (BMD) among those over 50 years old in the United States is 11% and 46%, respectively (3). Correspondingly, the direct medical cost caused by OP exceeds $20 billion annually, which is still increasing (4). China has a large population, and the incidence of osteoporosis in males and females over 50 years old is 10-20% and 30-40%, respectively (5). With the aging of the population, it can be predicted that the incidence of OP will continue to rise, resulting in multiple adverse effects such as pain, depression, and low quality of life. Hence, it is vital to systematically understand the risk factors of OP and carry out early prevention, diagnosis, and intervention.

A great deal of cooking oil fumes (COFs) produced are an essential source of household indoor air pollutants (6). Chinese cuisine is more likely to produce COFs. There are two paramount reasons. On the one hand, Chinese people generally cook by stir-frying. On the other hand, it is customary to wait until the oil is heated to smoke, and then add food and ingredients. Recently, several studies have confirmed that COFs increase the risk of various diseases, such as respiratory diseases, cardiovascular diseases, diabetes, cancer, and poor sleep quality (7–10). COFs contain particulate matter (PM), polycyclic aromatic hydrocarbons (PAHs), organic carbon (OC), volatile organic compounds (VOCs) and carbonyl compounds, etc (11–13). The particulate matter derived from COFs are predominantly of a diameter ≤ 2.5μm (PM_2.5_) (14–16). Previous studies have shown that air pollutants such as PAHs and PM_2.5_ can stimulate bone growth and inhibit bone resorption (17, 18). Cohort studies have also confirmed a potential relationship between long-term exposure to air pollution and OP (19, 20). The use of COFs extractor can effectively reduce the degree of indoor air pollution caused by COFs (21). Studies by Chen et al. presented that use of COFs extractors can significantly reduce the risk of lung cancer (22). It was reasonable to suspect that there was a potential relationship between long-term use of COFs extractors and BMD.

Consequently, this study attempted to explore the impact of non-use COFs extractors on BMD in population aged 45 years and older based on a prospective cross-sectional study in China. We hypothesized that using COFs extractors is an effective measure to prevent bone mass loss in the human body.

## Materials and methods

### Study design

This present study was a cross-sectional study within the framework of an ongoing prospective China Community-based Cohort of Osteoporosis (CCCO) (Clinical trials. gov., NCT02958020) (23, 24). Additionally, our research scheme has been approved by the Institutional Review Committee of Longhua Hospital, Shanghai University of Traditional Chinese Medicine (No. 2016LCSY065). This study was conducted in strict accordance with the Declaration of Helsinki, and all participants signed informed consent.

### Selection of study population

**Figure 1:**
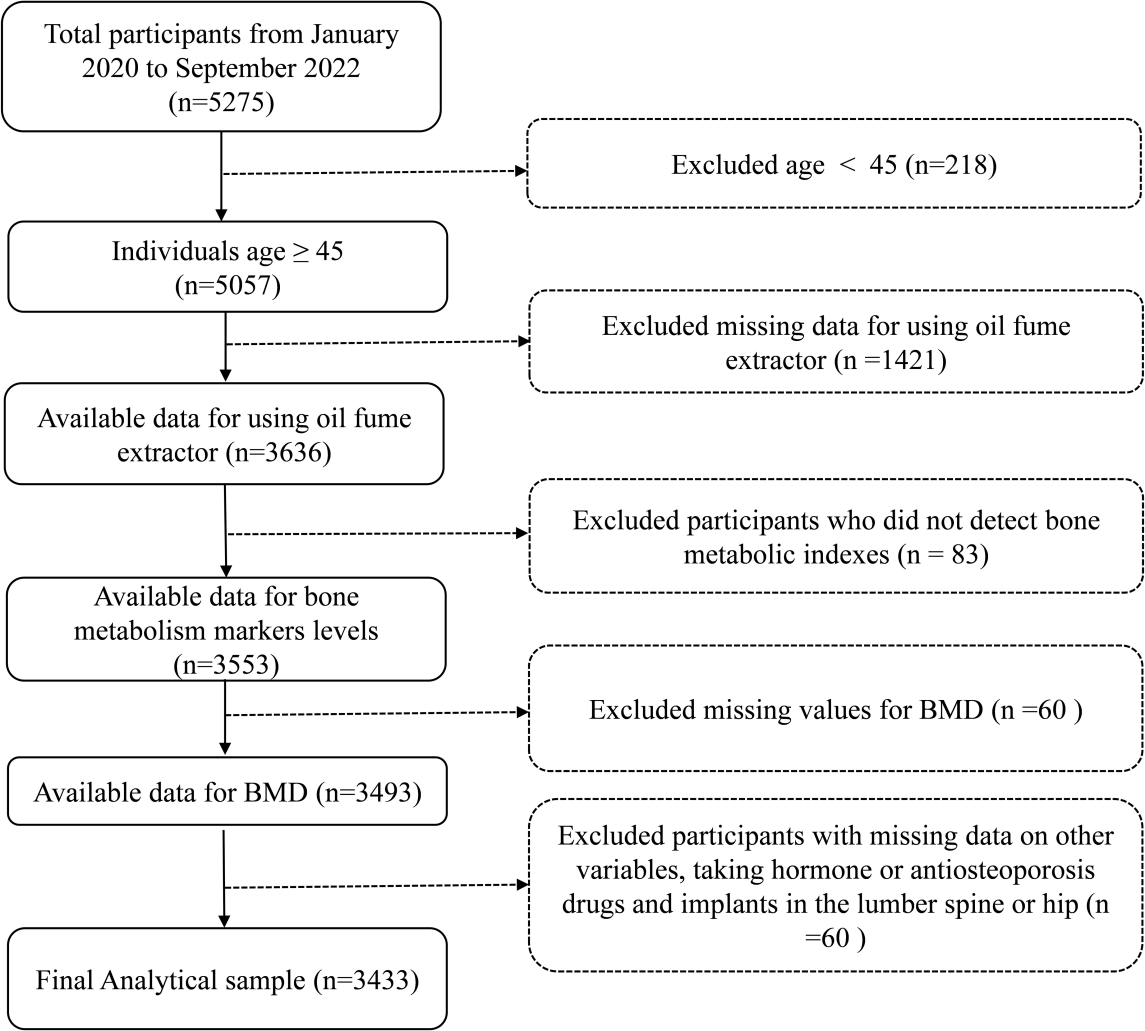
The study participant’s selection flow chart.

The screening of the study population was shown in **Figure 1**. The study population came from residents of Jiangxi province, China. Initially, 5275 participants were enrolled from January 2020 to September 2022. It is worth explaining that an age threshold of ≥ 45 years old selected to mitigate potential confounding influences, align with clinical practice relevance, and taking data availability into account. Thus, participants with under 45 years (n = 218) were excluded. Subsequently, participants with missing records on the use of COFs extractors (n =1421), missing bone metabolic indexes data (n = 83), missing BMD data (n = 60), missing data on other variables, taking hormone or antiosteoporosis drugs and fracture or implants in the lumber spine (LS) or hip (n = 60) were excluded in turn. Finally, 3433 participants were included in the analysis.

### Variable data

The independent variables of this study were whether participants used a COFs extractor. All participants were asked whether used a COFs extractor when cooking at home (yes/no). If yes, was it used on a long-term basis (≥ 3 years)? The individuals’ information about frequencies cooking at home (≥ once a day or < once a day), the type of cooking fuel used (clean fuel or solid fuel), and preference for frying food (yes or no) were also documented. All the aforementioned culinary-related data were obtained through a questionnaire.

The outcome variables were BMD (primary outcome) and bone metabolism markers (secondary outcome). The BMDs of each lumbar vertebra (L1, L2, L3, and L4), total LS, femoral neck (FN) and left hip (FN, trochanter, and intertrochanteric region combined with BMD) in all participants were quantified using dual energy X-ray absorptiometry (DXA, Hologic Discovery CI, Bedford, MA, USA). Calibration procedures routinely performed and calibration blocks provided to minimize measurement errors before each use of DXA.

Routine collection of fasting 5 ml venous blood from the median elbow vein of participants. The serum was isolated by centrifugation at the speed of 3,000 rpm for 15 minutes to detect the biochemical indexes of bone metabolism. Serum alkaline phosphatase (ALP) level was measured by continuous monitoring technique. The serum levels of osteocalcin (OST), N-terminal propeptide of type I collagen (PINP), and β-C-terminal telopeptide of type I collagen (β-CTX) were detected by electrochemiluminescence immunoassay. Serum creatinine content was determined by the creatinase method. The serum contents of calcium (Ca), phosphorus (P), and magnesium (Mg) were determined by the o-cresolphthalein-complex one method, phosphomolybdate ultraviolet colorimetry, and complex indicator method, respectively.

All participants were required to complete a paper questionnaire on OP under the guidance of trained professional clinicians. The contents of the questionnaire included baseline data, past medical history, family genetic history, living habits, eating habits, physical activity, and other modules. In this study, we extracted the required covariates according to the International Osteoporosis Foundation’s suggestion (25), including age, gender, and body mass index (BMI), self-reported marital status, smoking behavior, drinking behavior, history of parental osteoporosis, history of hypertension, history of hyperlipidemia, history of diabetes, history of knee osteoarthritis, history of gout, history of chronic obstructive pulmonary disease, history of chronic pharyngitis, and physical activity.

### Statistical analysis

Continuous variables were demonstrated as mean ± standard deviation and categorical variables were demonstrated as a percentage. The Mann-Whitney test and the Chi-square test were used to analyze the differences between the continuous variables and categorical variables, respectively. The multivariate linear regression models were used to evaluate the correlations of the non-use of fume extractors in family cooking with total LS, single LS, FN, hip BMD, and bone metabolism index level. The association between cooking frequency and total LS BMD was also further examined. In conformity with the Strengthening the Reporting of Observational Studies in Epidemiology (STROBE) statement guidelines published in 2007 (26), we have constructed the following three models: model 1, no covariates were adjusted; model 2, age, gender, and BMI were adjusted; model 3, age, gender, BMI, marital status, smoking behavior, drinking behavior, history of the parental OP, hypertension, hyperlipidemia, diabetes, knee osteoarthritis, gout, and physical activity were adjusted. Subsequently, subgroup analyses stratified by age, gender, smoking and drinking behavior were conducted, respectively. Furthermore, linear regression models were used to assess the independent relationship between cooking fuel type, food frying and total BMD of LS in participants using fume extractors.

All data were analyzed using R software (http://www.R-project.org, The R Foundation) and EmpowerStats software (http://empowwerstats.com/, X&Y Solutions, Inc., Boston, MA). *P* < 0.05 was considered statistically significant.

## Results

### Baseline characteristics of the study population

A total of 3433 participants were included in the final analyses, of which 2607 (75.93%) participants used fume extractors for home cooking on a long-term basis (group A) and 826 (24.06%) participants did not use fume extractors for home cooking (group B). There were significant differences in age, sex, marital status, smoking behavior, hypertension, and chronic obstructive pulmonary disease between the two groups (*P* < 0.05). The BMDs of L1, L2, L3, L4, total LS, and total hip in group A were higher than those in group B (all *P* < 0.05). However, the BMD of FN showed no significant difference between the two groups. Serum creatinine, PINP, β-CTX, and ALP levels were statistically significant between the two groups (all *P* < 0.05). In contrast, serum levels of OST, Serum P, Mg, and Ca were not significantly different between the two groups. Detailed baseline data for participants was shown in **Table 1**.

**Table 1:** Characteristics of the participants in a cross-sectional study.

Variables	Group A (n =2607)	Group B (n =826)	*P* value
Age, mean ± SD, year	61.44 ± 9.54	65.92 ± 9.05	**< 0.001**
Age, n (%)			**< 0.001**
45-60	1219(46.76%)	217 (26.27%)	
60-75	1155 (44.30%)	470 (56.90%)	
≥75	233 (8.94%)	139 (16.83%)	
Gender			**< 0.001**
male	563 (21.60%)	266 (32.20%)	
female	2044 (78.40%)	560 (67.80%)	
BMI, mean ± SD, m^2^/kg	23.28 ± 3.17	23.07 ± 3.60	0.103
Marital status, n (%)			**0.007**
Married	2473 (94.86%)	763 (92.37%)	
Others	134 (5.14%)	63 (7.63%)	
History of parental OP, n (%)			0.115
No	2526 (96.89%)	809 (97.94%)	
Yes	81 (3.11%)	17 (2.06%)	
Smoking behavior, n (%)			**0.015**
Never	2336(89.61%)	715 (86.56%)	
Current or past	271 (10.40%)	111 (13.44%)	
Drinking behavior, n (%)			0.994
Never	2228 (85.46%)	706 (85.47%)	
Current or past	379 (14.54%)	120 (14.53%)	
Hyperlipidemia, n (%)			0.162
No	2265 (86.88%)	733 (88.74%)	
Yes	342 (13.12%)	93 (11.25%)	
Hypertension, n (%)			**0.001**
No	1760 (67.51%)	508 (61.50%)	
Yes	847 (32.49%)	318 (38.50%)	
Knee osteoarthritis, n (%)			0.190
No	2319 (88.95%)	721 (87.29%)	
Yes	288 (11.05%)	105 (12.71%)	
Gout, n (%)			0.107
No	2548 (97.74%)	799 (96.73%)	
Yes	59 (2.26%)	27 (3.27%)	
Diabetes, n (%)			0.913
No	2367 (90.79%)	751 (90.92%)	
Yes	240 (9.21%)	75 (9.08%)	
Chronic pharyngitis, n (%)			
No	2343 (97.26%)	66 (2.74%)	0.184
Yes	66 (2.74%)	66 (2.74%)	
Chronic obstructive pulmonary disease, n (%)			0.006
No	2348 (97.55%)	59 (2.45%)	
Yes	59 (2.45%)	33 (4.39%)	
Physical activity, n (%)			0.050
Hardly any or mild exercise	694 (26.62%)	256 (30.99%)	
Moderate activities	794 (30.46%)	238 (28.81%)	
Vigorous activity	1119 (42.92%)	332 (40.19%)	
Frequencies of cooking, n (%)			0.093
≥once a day	1915 (73.46%)	631 (76.39%)	
< once a day	692 (26.54%)	195 (23.61%)	
L1 BMD, g/cm^2^	0.737 ± 0.167	0.713 ± 0.200	< **0.001**
L2 BMD, g/cm^2^	0.762 ± 0.186	0.722 ± 0.258	< **0.001**
L3 BMD, g/cm^2^	0.796 ± 0.192	0.749 ± 0.185	< **0.001**
L4 BMD, g/cm^2^	0.820 ± 0.231	0.780 ± 0.198	< **0.001**
Total LS BMD, g/cm^2^	0.783 ± 0.181	0.742 ± 0.165	< **0.001**
Total hip BMD, g/cm^2^	0.761 ± 0.173	0.741 ± 0.230	**0.010**
FN BMD, g/cm^2^	0.675 ± 0.195	0.665 ± 0.271	0.260
β-CTX, ng/ml	0.283 ± 0.163	0.269 ± 0.160	**0.037**
OST, ng/ml	15.614 ± 7.382	15.870 ± 7.103	0.381
PINP, ng/ml	61.271 ± 25.127	65.175 ± 27.018	< **0.001**
Serum creatinine	72.758 ± 23.360	77.967 ± 24.713	< **0.001**
ALP, U/L	83.607 ± 23.654	89.094 ± 27.890	< **0.001**
Serum P, mmol/L	1.307 ± 0.458	1.321 ± 0.507	0.447
Serum Mg, mmol/L	0.933 ± 0.072	0.938 ± 0.071	0.136
Serum Ca, mmol/L	2.346 ± 0.089	2.346 ± 0.103	0.999

ALP, alkaline phosphatase; β-CTX, β-C-terminal telopeptide of type I collagen, Ca, calcium, OST, osteocalcin, P, phosphorus, PINP, N-terminal propeptide of type I collagen, Mg, magnesium, OP, osteoporosis.

Mean ± SD for continuous variables: p value was calculated by the linear regression model. Number (proportion) for categorical variables: p value was calculated by the chi-square test. BMI, body mass index, LS, lumbar spine, FN, femoral neck, BMD, bone mineral density. Group A, use fume extractors, Group B, non-use fume extractors. The significance level is defined to be p < 0.05. Bold values indicate statistical difference.

### Association of non-use of fume extractors with BMD

The results of the linear regression model for the non-use of COFs extractors with total LS, total hip, and FN BMD showed that there was a significant negative correlation between the non-use of COFs extractors and total LS BMD (model 3: β = -0.024, 95% CI, -0.036, -0.012, *p* < 0.001). Nevertheless, there was no correlation between the non-use of fume extractors and FN BMD (model 3: β = -0.014, 95% CI: -0.014, 0.019, *p* = 0.762) and the total BMD of the hip (model 3: β = -0.004, 95% CI: -0.018, 0.010, *p* = 0.591) (**Table 2**).

**Table 2:** Associations of the non-use of fume extractors with total LS, femoral neck, and total hip BMD.

Outcome	Model 1, β (95% CI) *P*	Model 2, β (95% CI) *P*	Model 3, β (95% CI) *P*
Total LS BMD	-0.041 (-0.054, -0.027) < **0.001**	-0.025 (-0.038, -0.013) < **0.001**	-0.024 (-0.036, -0.012) < **0.001**
Femoral neck BMD	-0.010 (-0.027, 0.007) 0.260	-0.001 (-0.016, 0.017) 0.937	0.003 (-0.014, 0.019) 0.762
Total hip BMD	-0.019 (-0.034, -0.005) < **0.001**	-0.006 (-0.019, 0.008) 0.427	-0.004 (-0.018, 0.010) 0.591

LS, lumbar spine; BMD, bone mineral density; OP, osteoporosis. Model 1: no covariates were adjusted; model 2: age, gender, and BMI were adjusted; model 3: age, gender; BMI, marital status, smoking behavior, drinking behavior, history of the parental OP, hypertension, hyperlipidemia, diabetes, knee osteoarthritis, gout, and physical activity. The significance level is defined to be p < 0.05. Bold values indicate statistical difference.

The results of the multivariate linear regression model between the non-use of COFs extractors and single LS BMD presented that the non-use of fume extractor was negatively correlated with L2 (model 3: β = -0.023, 95% CI: -0.038, -0.008, *p* = 0.002), L3 (model 3: β = -0.028, 95% CI: -0.042, -0.015, *p* < 0.001), L4 (model 3: β = -0.026, 95% CI: -0.043, 0.010, *p* = 0.002) BMD. However, the non-use of the fume extractor was not associated with L1 BMD (model 3: β = -0.003, 95% CI: -0.018, -0.012, *p* = 0.672) (**Table 3**).

**Table 3:** Relationship of non-use of fume extractors with individual total LS BMD.

Outcome	Model 1, β (95% CI) *P*	Model 2, β (95% CI) *P*	Model 3, β (95% CI) *P*
L1 BMD	-0.024 (-0.038, -0.011) **< 0.001**	-0.013 (-0.026, -0.000) 0.047	-0.012 (-0.025, 0.001) 0.067
L2 BMD	-0.040 (-0.056, -0.024) < **0.001**	-0.024 (-0.039, -0.009) **0.002**	-0.023 (-0.038, -0.008) **0.002**
L3 BMD	-0.048 (-0.063, -0.033) < **0.001**	-0.030 (-0.043, -0.017) < **0.001**	-0.028 (-0.042, -0.015) < **0.001**
L4 BMD	-0.040 (-0.058, -0.023) < **0.001**	-0.027 (-0.044, -0.011) < **0.001**	-0.026 (-0.043, -0.010) **0.002**

LS, lumbar spine, BMD, bone mineral density, OP, osteoporosis. Model 1: no covariates were adjusted; model 2: age, gender, and BMI were adjusted; model 3: age, gender; BMI, marital status, smoking behavior, drinking behavior, history of the parental OP, hypertension, hyperlipidemia, diabetes, knee osteoarthritis, gout, and physical activity. The significance level is defined to be p < 0.05. Bold values indicate statistical difference.

### Independent association of cooking frequencies with total LS BMD

In the group of using of fume extractors, it was observed that individuals cooking frequency ≥ once a day had a lower LS BMD when compared to those cooking less than once a day (model 3: β = -0.067, 95% CI: -0.082, -0.052, *p* < 0.001), adjusting for confounding variables. Similar association existed in the group of non-use of fume extractors (**Table 4**).

**Table 4:** Association of cooking frequencies with total LS BMD.

Groups	Cooking frequencies	Model 1, β (95% CI) *P*	Model 2, β (95% CI) *P*	Model 3, β (95% CI) *P*
Use fume extractors	≥once a day	Reference	Reference	Reference
	< once a day	0.122 (0.107, 0.137) **<0.001**	0.068 (0.053, 0.083) **<0.001**	0.067 (0.052, 0.082) **<0.001**
Non-use fume extractors	≥once a day	Reference	Reference	Reference
	< once a day	0.095 (0.070, 0.121) **<0.001**	0.043 (0.017, 0.068) **0.001**	0.049 (0.022, 0.075) **<0.001**

LS, lumbar spine, BMD, bone mineral density, OP, osteoporosis. Model 1: no covariates were adjusted; model 2: age, gender; and BMI were adjusted; model 3: age, gender, BMI, marital status, smoking behavior, drinking behavior, history of the parental OP, hypertension, hyperlipidemia, diabetes, knee osteoarthritis, gout, and physical activity. The significance level is defined to be p < 0.05. Bold values indicate statistical difference.

### Stratification analysis

In the subgroup analysis stratified by age, the adverse effects of not using fume extractors were only observed in the population aged 45-60 years (β = -0.051, 95% CI: -0.075, -0.027, *p* < 0.001). In the subgroup analysis stratified by gender, smoking behavior, and drinking behavior, the detrimental impact of not using fume extractors appeared to be more significant among males (males, β = -0.028, 95%CI: -0.050, -0.006, *p* = 0.014; female, β = -0.018, 95%CI: -0.032, -0.003, *p* = 0.016), smokers (smokers, β = -0.042, 95%CI: -0.077, -0.006, p = 0.022; never smoking, β = -0.019, 95%CI: -0.032, -0.006, *p* = 0.004), and drinkers (drinker, β = -0.039, 95%CI: -0.070, -0.007, *p* = 0.016; never drinking, β = -0.020, 95%CI: -0.033, -0.006, *p* = 0.004) (**Table 5**).

**Table 5:** The results of stratification.

Stratified variable	Total LS BMD		
	Model 1, β (95% CI) *P*	Model 2, β (95% CI) *P*	Model 3, β (95% CI) *P*
Age			
45-60 years	-0.031 (-0.056, -0.006) **0.016**	-0.051 (-0.075, -0.027) < **0.001**	-0.051 (-0.075, -0.027) < **0.001**
60-75 years	-0.008 (-0.026, 0.009) 0.350	-0.013 (-0.028, 0.002) 0.095	-0.009 (-0.024, 0.006) 0.245
≥75 years	-0.022 (-0.059, 0.015) 0.241	-0.006 (-0.038, 0.026) 0.706	0.005 (-0.027, 0.036) 0.772
Gender			
male	-0.047 (-0.070, -0.024) < **0.001**	-0.034 (-0.056, -0.012) **0.002**	-0.028 (-0.050, -0.006) **0.014**
female	-0.053 (-0.069, -0.036) < **0.001**	-0.018 (-0.032, -0.003) **0.017**	-0.018 (-0.032, -0.003) **0.016**
Smoking behavior			
Never	-0.040 (-0.055, -0.026) < **0.001**	-0.020 (-0.033, -0.007) **0.003**	-0.019 (-0.032, -0.006) **0.004**
Current or past	-0.062 (-0.099, -0.025) **0.011**	-0.049(-0.085, -0.014) 0.007	-0.042 (-0.077, -0.006) **0.022**
Drinking behavior			
Never	-0.040 (-0.055, -0.025) < **0.001**	-0.021 (-0.035, -0.008) **0.002**	-0.020 (-0.033, -0.006) **0.004**
Current or past	-0.046 (-0.079, -0.013) **0.007**	-0.042 (-0.073, -0.010) **0.009**	-0.039 (-0.070, -0.007) **0.016**

LS, lumbar spine, BMD, bone mineral density, OP, osteoporosis. Model 1: no covariates were adjusted; model 2: age, gender, and BMI were adjusted; model 3: age, gender; BMI, marital status, smoking behavior, drinking behavior, history of the parental OP, hypertension, hyperlipidemia, diabetes, knee osteoarthritis, gout, and physical activity. The significance level is defined to be p < 0.05.

*In the subgroup analysis stratified, the model is not adjusted for the stratification variable itself.Bold values indicate statistical difference.

### Association of non-use of fume extractors with bone metabolic markers level

The results of the linear regression model without using fume extractors and bone metabolic markers showed significant positive associations of non-use of fume extractors with PINP (model 3: β = 4.363, 95% CI, 2.371, 6.356, *p* < 0.001) and ALP (model 3: β = 4.555, 95% CI, 2.593, 6.517, *p* < 0.001) levels. There was no correlation between the non-use of fume extractors and β-CTX and other markers after adjusting the factors (**Table 6**).

**Table 6:** Associations of the non-use of fume extractors with bone metabolic markers level.

Bone metabolic markers	Model 1, β (95% CI) *P*	Model 2, β (95% CI) *P*	Model 3, β (95% CI) *P*
β-CTX	-0.013 (-0.026, -0.001) **0.037**	-0.002 (-0.015, 0.011) 0.735	-0.003 (-0.016, 0.009) 0.615
OST	0.256 (-0.316, 0.829) 0.381	0.430 (-0.140, 0.999) 0.139	0.366 (-0.195, 0.928) 0.201
PINP	3.904 (1.901, 5.907) < **0.001**	4.595 (2.588, 6.602) < **0.001**	4.363 (2.371, 6.356) < **0.001**
Serum creatinine	5.210 (3.356, 7.064) < **0.001**	0.971 (-0.683, 2.624) 0.250	1.007 (-0.640, 2.655) 0.231
ALP	5.488 (3.552, 7.424) < **0.001**	4.584 (2.624, 6.544) < **0.001**	4.555 (2.593, 6.517) < **0.001**
Serum P	0.014 (-0.023, 0.051) 0.447	0.014 (-0.023, 0.051) 0.459	0.014 (-0.023, 0.052) 0.451
Serum Mg	0.004 (-0.001, 0.010) 0.136	-0.001 (-0.007, 0.004) 0.673	-0.001 (-0.007, 0.004) 0.689
Serum Ca	-0.000 (-0.007, 0.007) 0.999	0.003 (-0.004, 0.010) 0.403	0.004 (-0.003, 0.011) 0.273

ALP, alkaline phosphatase; β-CTX, β-C-terminal telopeptide of type I collagen, Ca, calcium; OST, osteocalcin, P, phosphorus; PINP, N-terminal propeptide of type I collagen, Mg, magnesium, OP, osteoporosis.

Model 1: no covariates were adjusted; model 2: age, gender, and BMI were adjusted; model 3: age, gender; BMI, marital status, smoking behavior, drinking behavior, history of the parental OP, hypertension, hyperlipidemia, diabetes, knee osteoarthritis, gout, and physical activity. The significance level is defined to be p < 0.05. Bold values indicate statistical difference.

### Association of cooking fuel type and food frying with total LS BMD

Among the people who use oil fume extractors, 2294 people have recorded cooking fuel types, including 2088 participants who use clean fuel (natural gas, liquefied gas, or electricity), 206 participants who use solid fuel (coal). Compared with clean fuel, the results of model indicated that there was a negative correlation between solid fuel use and total LS BMD (model 3: β = -0.049, 95%CI: - 0.074, -0.024, *p* < 0.001).

There were 2,692 participants using fume extractors who had records of frying food, including 2,160 who often liked fried food and 532 who never fried food. The results of model indicated that there was no significant correlation between frying food and total LS BMD (model 3: β = -0.004, 95%CI: -0.019, 0.011, *p* = 0.607) (**Table 7**).

**Table 7:** Association of fuel type and food frying with LS BMD in the population using fume extractors.

	Model 1 (β, 95% CI, P)	Model 2 (β, 95% CI, P)	Model 3 (β, 95% CI, P)
Fuel type			
Clean fuel (n=2088)	Reference	Reference	Reference
Solid fuel (n=206)	-0.068 (-0.094, -0.043) < **0.001**	-0.039 (-0.061, -0.016) < **0.001**	-0.045 (-0.067, -0.022) < **0.001**
Food frying			
No (n=2160)	Reference	Reference	Reference
Yes (n=532)	-0.019 (-0.036, -0.002) **0.032**	-0.001 (-0.016, 0.014) 0.918	-0.004 (-0.019, 0.011) 0.607

LS, lumbar spine, BMD, bone mineral density, OP, osteoporosis. Model 1: no covariates were adjusted; model 2: age, gender, and BMI were adjusted; model 3: age, gender; BMI, marital status, smoking behavior, drinking behavior, history of parental OP, hypertension, hyperlipidemia, diabetes, knee osteoarthritis, gout and physical activity. Bold values indicate statistical difference.

## Discussion

In this prospective cross-sectional study of China, we systematically assessed the relationship between COFs extractors use and total LS, FN, and total hip BMD in adults ≥ 45 age. The results confirmed that long-term use of COFs extractors is an effective measure to prevent bone loss, particularly among groups of 45-60 years old, males, smokers (current or past smokers) and drinkers (current or past smokers). The higher the cooking frequency, the greater the decrease in LS BMD. Additionally, we found that solid fuel use was associated with lower total LS BMD among COFs extractor users.

Long-term exposure to air pollution was a significant independent risk factor for bone mass decline (27). A study from rural areas of Henan Province in China demonstrated that PM content in the air was positively correlated with OP (19). A study based on the UK Biobank manifested that people exposed to PM_2.5_, NO_2_, and NO_X_ increased the incidence of OP (28). A large national cohort study in South Korea proved that long-term exposure to SO_2_ was associated with an increased risk of osteoporotic fractures (29). Aldehydes were one of the environmental pollutants. Gu et al. deemed that mixed aldehydes can significantly reduce BMD in males (30). Duan et al. considered that 2-hydroxy fluorene was associated with increased odds of OP (31). The above evidence indicates that air pollution was related to lower BMD. COFs were the main culprit of indoor air pollution. Most harmful substances in COFs are similar to outdoor air pollution, such as PM (31).

**Figure 2:**
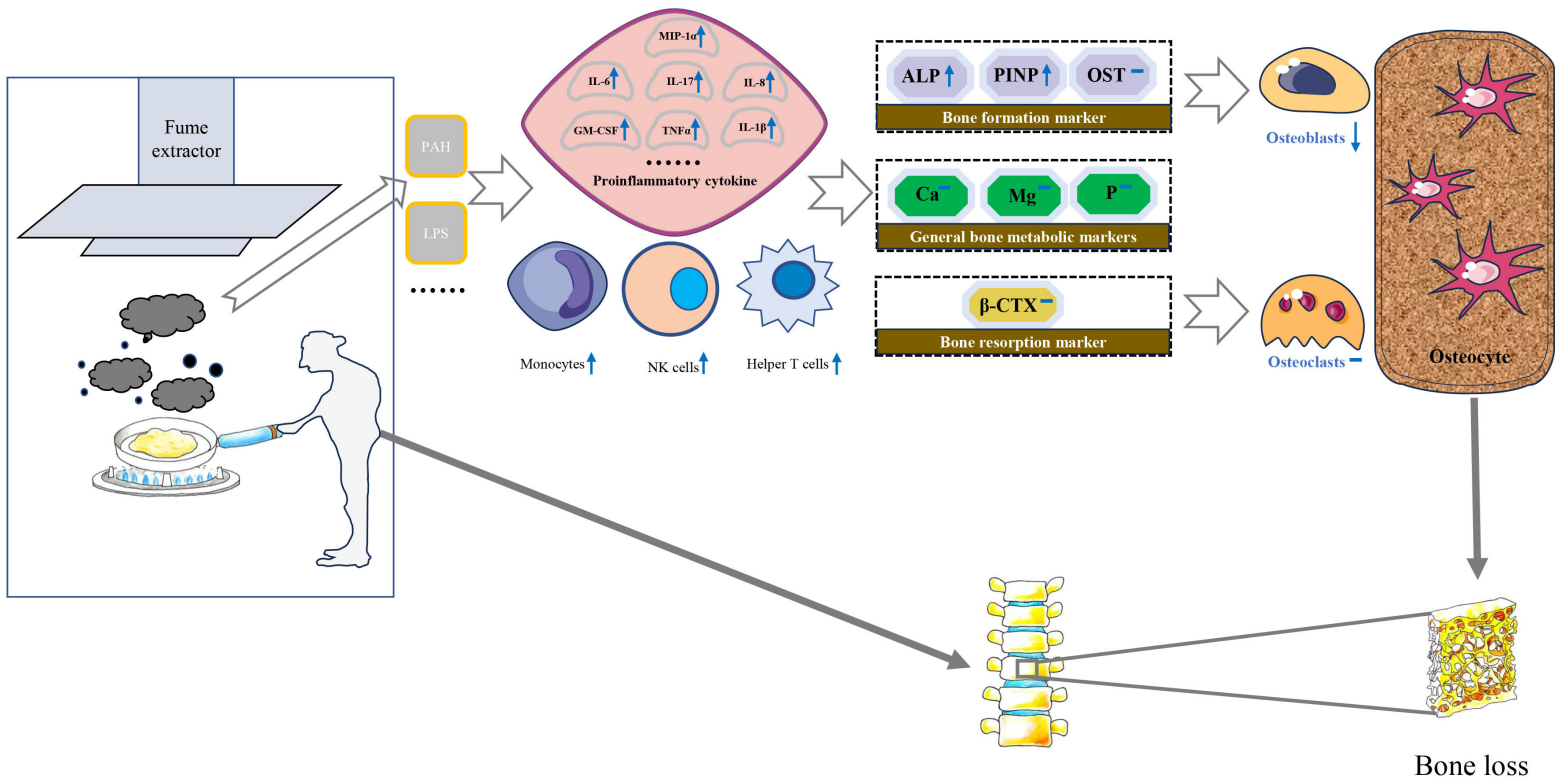
Possible molecular and cellular mechanisms for the impact of cooking oil fume on bone mineral density. IL, Interleukin; MCSF, Macrophage colony-stimulating factor; TNF-α, Tumor necrosis factor-alpha; PAH, polycyclic aromatic hydrocarbons; LPS, lipopolysaccharide; MIP-1α, Macrophage inflammatory protein 1α; ALP, alkaline phosphatase; β-CTX, β-C-terminal telopeptide of type I collagen; Ca, calcium; OST, osteocalcin; P, phosphorus; PINP, N-terminal propeptide of type I collagen; Mg, magnesium.

Biologically, exposure to high concentrations of PM may stimulate inflammatory responses in alveolar macrophages and airway epithelial cells, which significantly increase the levels of serum monocytes, NK cells, and helper T cells and induce the expression of proinflammatory cytokines including tumor necrosis factor α, monocyte chemoattractant protein-1, interleukin-8, macrophage inflammatory protein-1 α, IL-6, IL-1 β and granulocyte-macrophage colony-stimulating factor (32–34). Subsequently, some inflammatory factors may increase receptor activators of nuclear factor κ B expression in osteoclast precursors and macrophage colony-stimulating factor expression in stromal cells. Eventually, an imbalance of osteoblasts and osteoclasts leads to bone loss (18, 35) (**Figure 2**).

The fume extractors can effectively discharge the lampblack produced during cooking. Notwithstanding, in many rural areas of China, the use of fume extractors is not all widespread. Currently, few researchers have investigated the relationship between the use of fume extractors and human BMD. Thereby, we conducted a cross-sectional study of southern China. Our study verified that using fume extractors in family cooking can effectively prevent the loss of total LS BMD aged 45-60 in southern China, which might provide evidence for the local government to formulate valuable policies. We recommend that the kitchen be equipped with a fume extractor and use this device when cooking to reduce the unfavorable impact of COFs on public bones. Incidentally, we found no evidence of an association between the use of a fume extractor and hip or femoral neck BMD. Although there is no direct experimental evidence for the susceptibility of different bones to air pollution, Ranzani et al. believed that trabecular bone was more affected than cortical bone (36). Further study on the effect of different parts of BMD is necessary.

As for the relationship between the use of COFs extractors and single LS BMD, the results of L2, L3, and L4 were consistent with the overall lumbar BMD except for L1. This may involve the biomechanical effects of the LS. The L1 segment has a large range of motion, and it is the transition point from thoracic kyphosis to lumbar kyphosis. The L1 trabecular bone tissue possesses distinctive characteristics, serving as a pivotal site in vertebral fractures. Among the fragility fractures of the lumbar vertebrae, L1 has the highest incidence (37). For this reason, the effect COFs on L1 was different from other LS.

What is worth mentioning was the result of the hierarchical analysis. After the adjustment of the model, the total LS BMD of people aged 45-60 without using fume extractors were significantly lower than that of using fume extractor, and that phenomenon disappeared over 60. One possible explanation was that the activity of osteoclasts and osteoblasts of the elderly decreases with age (38, 39). The adverse effect of COFs was less prominent in the competition between osteoclasts and osteoblasts with reduced activity (40). In the stratification analysis of gender, although the negative correlation between the non-use of fume extractor and total LS BMD was present in both males and females, bone loss was more severe in males. This result was different from previous studies in Taiwan (41). It was speculated that the women in this study are all 45 years old and older, most of whom were postmenopausal. Postmenopausal women lack the combination of hormone factors and particulate matter. Moreover, total LS BMD declines in smokers were more severe than in non-smokers, which was consistent with previous studies (42, 43). Several toxic components in tobacco smoke were the same as those in particulates. Nicotine and other toxic components in tobacco smoke can promote osteoclast differentiation, which was the superimposed harmful factor of COFs (44). Similarly, total LS BMD declines in drinkers were more severe than in non-drinkers. The effect of alcohol intake on BMD has always been controversial, and studies had shown that the effect of alcohol on BMD depends on the amount consumed (45). As a consequence, this study strongly recommends that certain groups of people aged 45-60 years, males, smokers, or drinkers focus on using fume extractors to prevent loss of LS BMD while cooking.

The user of solid fuel had lower total LS BMD compared to the clean fuel in the population of using fume extractors. A cohort study covering 28 villages in southern India reported that black carbon was associated with lower levels of BMD in the hips and LS, although not statistically significant (36). On the one hand, the primary mechanism by which the toxic components produced by black charcoal burning affect bone loss may stimulate pro-inflammatory cytokines to induce RANKL secretion (46). On the other hand, black carbon exposure is negatively correlated with parathyroid hormone levels (47). Our study revealed that the behavior of food frying had no significant effect on total LS BMD while using fume extractors.

The detection of serum biochemical markers can indirectly reflect the status of bone metabolism in the whole body to some extent (48). Bone turnover markers are divided into bone formation markers and bone resorption markers. PINP, ALP and OST belong to bone formation markers. ALP was an extracellular enzyme secreted by osteoblasts. Its physiological function was to hydrolyze phosphate and pyrophosphate during osteogenesis (49). When bone mineralization was blocked, osteoblasts would synthesize a large amount of ALP. In addition, type I collagen was the only type of collagen in mineralized bone in the human body. The amino terminal additional peptide removed from procollagen was called PINP. PINP is recognized as the best marker for evaluating bone formation (50). The increased expression of PINP indicates that the synthesis rate of type I collagen was fast and bone turnover is active (51). β-CTX primarily serves as an indicator of osteoclast activity and bone resorption levels, thereby indirectly gauging the extent of osteoporosis (52). Liu et al. observed a significant positive correlation between exposure to air pollutants and bone calcium and β-CTX levels of children (53). Conversely, the investigation led by Feizabad et al. yielded no noteworthy differentiation in β-CTX levels between adolescents inhabiting polluted and unpolluted locales (54). A basic study found that rats exposed to air pollutants had significantly higher ALP levels than the control group (55). There was limited research on the correlation between air pollution and PINP, which made it challenging to compare our current findings with previous research. In our study, serum levels of PINP and ALP were significantly lower in individuals with using fume extractors compared to without using fume extractors. This condition is not reflected in β-CTX and other bone metabolic markers. Therefore, we speculate that the biological mechanism of BMD loss caused by COFs exposure may destroy the balance of bone remodeling mainly by affecting the process of bone formation (**Figure 2**).

To the best of our knowledge, this study is the first to investigate the association between household fume extractors use and BMD based on cross-sectional population. Admittedly, there are certain limitations in this study. Firstly, the exposure variables in this study were based on self-reported results, which memory errors of a small number of participants may have been biased. Secondly, the exposure factors in this study were qualitative, while the content of COFs was not quantified. We will further refine the COFs content in the ongoing cohort study. Last but not least, there was the bias caused by certain unavoidable potential confounding factors, such as the type of COFs.

## Conclusion

In a nutshell, this study verified that the use of fume extractors was an efficacious measure to prevent bone loss. The use of fume extractors may keep serum PINP and ALP at a low level. For the sake of public bone health, we recommended that people should install a fume extractor in the kitchen and use it routinely when cooking. Particularly, the specific population aged 45-60, males, smokers, or drinkers are required to follow this recommendation. In addition, clean fuel should be used as much as possible when cooking to reduce the adverse impact on LS BMD.

## Data availability statement

The original contributions presented in the study are included in the article/supplementary material. Further inquiries can be directed to the corresponding author.

## Ethics statement

The cross-sectional study has been approved by the Institutional Review Committee of Longhua Hospital, Shanghai University of Traditional Chinese Medicine (No. 2016LCSY065). This study was conducted in strict accordance with the Declaration of Helsinki, and all participants signed informed consent.

## Author contributions

HZ: Data curation, Formal analysis, Investigation, Methodology, Software, Visualization, Writing – original draft. BShi: Data curation, Investigation, Methodology, Software, Writing – original draft. CY: Data curation, Formal analysis, Investigation, Methodology, Software, Supervision, Writing – original draft. CH: Data curation, Investigation, Methodology, Writing – original draft. TH: Data curation, Investigation, Writing – original draft. ZL: Data curation, Investigation, Writing – review & editing. WZhu: Data curation, Formal analysis, Investigation, Methodology, Software, Validation, Visualization, Writing – original draft. WZho: Data curation, Investigation, Writing – review & editing. HX: Data curation, Investigation, Software, Validation, Writing – original draft. JJ: Data curation, Investigation, Software, Writing – original draft. FC: Data curation, Investigation, Writing – original draft. YC: Data curation, Investigation, Writing – original draft. PS: Data curation, Investigation, Writing – original draft. XZ: Data curation, Investigation, Writing – review & editing. ZY: Data curation, Investigation, Writing – review & editing. JW: Data curation, Methodology, Formal analysis, Supervision, Writing – review & editing. BShu: Writing – review & editing, Project administration, Supervision. QL: Supervision, Writing – review & editing, Validation. QS: Supervision, Writing – review & editing, Conceptualization. CX: Conceptualization, Supervision, Writing – review & editing, Funding acquisition. DT: Conceptualization, Supervision, Writing – review & editing, Project administration, Resources, Funding acquisition. YW: Conceptualization, Project administration, Resources, Supervision, Writing – review & editing, Funding acquisition.

## References

[1] Johnston CB Dagar M. Osteoporosis in older adults. Med Clinics North America (2020) 104(5):873–84. doi: 10.1016/j.mcna.2020.06.004 32773051

[2] Consensus development conference: diagnosis, prophylaxis, and treatment of osteoporosis. Am J Med (1993) 94(6):646–50. doi: 10.1016/0002-9343(93)90218-e 8506892

[3] Looker AC Sarafrazi Isfahani N Fan B Shepherd JA. Trends in osteoporosis and low bone mass in older US adults, 2005-2006 through 2013-2014. Osteoporosis Int J established as result cooperation between Eur Foundation Osteoporosis Natl Osteoporosis Foundation USA (2017) 28(6):1979–88. doi: 10.1007/s00198-017-3996-1 PMC7891684 28315954

[4] Dempster DW. Osteoporosis and the burden of osteoporosis-related fractures. Am J managed Care (2011) 17 Suppl 6:S164–9. 21761955

[5] Yu F Xia W. The epidemiology of osteoporosis, associated fragility fractures, and management gap in China. Arch osteoporosis (2019) 14(1):32. doi: 10.1007/s11657-018-0549-y 30848398

[6] Straif K Baan R Grosse Y Secretan B El Ghissassi F Cogliano V. Carcinogenicity of household solid fuel combustion and of high-temperature frying. Lancet Oncol (2006) 7(12):977–8. doi: 10.1016/S1470-2045(06)70969-X 17348122

[7] Wei F Nie G Zhou B Wang L Ma Y Peng S. Association between Chinese cooking oil fumes and sleep quality among a middle-aged Chinese population. Environ pollut (Barking Essex 1987) (2017) 227:543–51. doi: 10.1016/j.envpol.2017.05.018 28501768

[8] Hou X Mao Z Song X Kang N Zhang C Li R. Kitchen ventilation alleviated adverse associations of domestic fuel use and long-duration cooking with platelet indices as biomarkers of cardiovascular diseases. Sci Total Environ (2022) 834:155341. doi: 10.1016/j.scitotenv.2022.155341 35452724

[9] Hou J Sun H Zhou Y Zhang Y Yin W Xu T. Environmental exposure to polycyclic aromatic hydrocarbons, kitchen ventilation, fractional exhaled nitric oxide, and risk of diabetes among Chinese females. Indoor air (2018) 28(3):383–93. doi: 10.1111/ina.12453 29444361

[10] Jia PL Zhang C Yu JJ Xu C Tang L Sun X. The risk of lung cancer among cooking adults: a meta-analysis of 23 observational studies. J Cancer Res Clin Oncol (2018) 144(2):229–40. doi: 10.1007/s00432-017-2547-7 PMC11813306 29164315

[11] Singh A Chandrasekharan Nair K Kamal R Bihari V Gupta MK Mudiam MK. Assessing hazardous risks of indoor airborne polycyclic aromatic hydrocarbons in the kitchen and its association with lung functions and urinary PAH metabolites in kitchen workers. Clinica chimica acta; Int J Clin Chem (2016) 452:204–13. doi: 10.1016/j.cca.2015.11.020 26616733

[12] Sjaastad AK Jørgensen RB Svendsen K. Exposure to polycyclic aromatic hydrocarbons (PAHs), mutagenic aldehydes and particulate matter during pan frying of beefsteak. Occup Environ Med (2010) 67(4):228–32. doi: 10.1136/oem.2009.046144 20164502

[13] Yao Z Li J Wu B Hao X Yin Y Jiang X. Characteristics of PAHs from deep-frying and frying cooking fumes. Environ Sci pollut Res Int (2015) 22(20):16110–20. doi: 10.1007/s11356-015-4837-4 26066859

[14] Ding L Sui X Yang M Zhang Q Sun S Zhu F. Toxicity of cooking oil fume derived particulate matter: Vitamin D(3) protects tubule formation activation in human umbilical vein endothelial cells. Ecotoxicol Environ Saf (2020) 188:109905. doi: 10.1016/j.ecoenv.2019.109905 31706245

[15] Ke Y Huang L Xia J Xu X Liu H Li YR. Comparative study of oxidative stress biomarkers in urine of cooks exposed to three types of cooking-related particles. Toxicol Lett (2016) 255:36–42. doi: 10.1016/j.toxlet.2016.05.017 27208482

[16] Li YC Qiu JQ Shu M Ho SSH Cao JJ Wang GH. Characteristics of polycyclic aromatic hydrocarbons in PM(2.5) emitted from different cooking activities in China. Environ Sci pollut Res Int (2018) 25(5):4750–60. doi: 10.1007/s11356-017-0603-0 29198025

[17] Guo J Huang Y Bian S Zhao C Jin Y Yu D. Associations of urinary polycyclic aromatic hydrocarbons with bone mass density and osteoporosis in U.S. adults, NHANES 2005-2010. Environ pollut (Barking Essex 1987) (2018) 240:209–18. doi: 10.1016/j.envpol.2018.04.108 29738949

[18] Prada D López G Solleiro-Villavicencio H Garcia-Cuellar C Baccarelli AA. Molecular and cellular mechanisms linking air pollution and bone damage. Environ Res (2020) 185:109465. doi: 10.1016/j.envres.2020.109465 32305664 PMC7430176

[19] Qiao D Pan J Chen G Xiang H Tu R Zhang X. Long-term exposure to air pollution might increase prevalence of osteoporosis in Chinese rural population. Environ Res (2020) 183:109264. doi: 10.1016/j.envres.2020.109264 32311909

[20] Yang Y Li R Cai M Wang X Li H Wu Y. Ambient air pollution, bone mineral density and osteoporosis: Results from a national population-based cohort study. Chemosphere (2023) 310:136871. doi: 10.1016/j.chemosphere.2022.136871 36244420

[21] Feng S Shen X Hao X Cao X Li X Yao X. Polycyclic and nitro-polycyclic aromatic hydrocarbon pollution characteristics and carcinogenic risk assessment of indoor kitchen air during cooking periods in rural households in North China. Environ Sci pollut Res Int (2021) 28(9):11498–508. doi: 10.1007/s11356-020-11316-8 33123888

[22] Chen TY Fang YH Chen HL Chang CH Huang H Chen YS. Impact of cooking oil fume exposure and fume extractor use on lung cancer risk in non-smoking Han Chinese women. Sci Rep (2020) 10(1):6774. doi: 10.1038/s41598-020-63656-7 32317677 PMC7174336

[23] Wang J Chen L Zhang Y Li CG Zhang H Wang Q. Association between serum vitamin B(6) concentration and risk of osteoporosis in the middle-aged and older people in China: a cross-sectional study. BMJ Open (2019) 9(7):e028129. doi: 10.1136/bmjopen-2018-028129 PMC6615830 31278103

[24] Wang J Shu B Tang DZ Li CG Xie XW Jiang LJ. The prevalence of osteoporosis in China, a community based cohort study of osteoporosis. Front Public Health (2023) 11:1084005. doi: 10.3389/fpubh.2023.1084005 36875399 PMC9978786

[25] Kanis JA Delmas P Burckhardt P Cooper C Torgerson D. Guidelines for diagnosis and management of osteoporosis. Eur Foundation Osteoporosis Bone Disease. Osteoporosis Int J established as result cooperation between Eur Foundation Osteoporosis Natl Osteoporosis Foundation USA (1997) 7(4):390–406. doi: 10.1007/BF01623782 9373575

[26] von Elm E Altman DG Egger M Pocock SJ Gøtzsche PC Vandenbroucke JP. The Strengthening the Reporting of Observational Studies in Epidemiology (STROBE) statement: guidelines for reporting observational studies. Lancet (2007) 370(9596):1453–7. doi: 10.1016/S0140-6736(07)61602-X 18064739

[27] Torkashvand J Jonidi Jafari A Pasalari H Shahsavani A Oshidari Y Amoohadi V. The potential osteoporosis due to exposure to particulate matter in ambient air: Mechanisms and preventive methods. J Air Waste Manage Assoc (1995) (2022) 72(9):925–34. doi: 10.1080/10962247.2022.2085820 35653555

[28] Xu C Weng Z Liu Q Xu J Liang J Li W. Association of air pollutants and osteoporosis risk: The modifying effect of genetic predisposition. Environ Int (2022) 170:107562. doi: 10.1016/j.envint.2022.107562 36228550

[29] Heo S Kim H Kim S Choe SA Byun G Lee JT. Associations between long-term air pollution exposure and risk of osteoporosis-related fracture in a nationwide cohort study in South Korea. Int J Environ Res Public Health (2022) 19(4):2404. doi: 10.3390/ijerph19042404 35206592 PMC8872590

[30] Gu L Wang Z Liu L Luo J Pan Y Sun L. Association between mixed aldehydes and bone mineral density based on four statistical models. Environ Sci Pollut Res Int (2023) 30(11):31631–46. doi: 10.1007/s11356-022-24373-y 36450965

[31] Duan W Meng X Sun Y Jia C. Association between polycyclic aromatic hydrocarbons and osteoporosis: data from NHANES, 2005-2014. Arch osteoporosis (2018) 13(1):112. doi: 10.1007/s11657-018-0527-4 30334129

[32] Pope CA 3rd Bhatnagar A McCracken JP Abplanalp W Conklin DJ O'Toole T. Exposure to fine particulate air pollution is associated with endothelial injury and systemic inflammation. Circ Res (2016) 119(11):1204–14. doi: 10.1161/CIRCRESAHA.116.309279 PMC5215745 27780829

[33] Alfaro-Moreno E Torres V Miranda J Martínez L García-Cuellar C Nawrot TS. Induction of IL-6 and inhibition of IL-8 secretion in the human airway cell line Calu-3 by urban particulate matter collected with a modified method of PM sampling. Environ Res (2009) 109(5):528–35. doi: 10.1016/j.envres.2009.02.010 19304283

[34] Becker S Dailey L Soukup JM Silbajoris R Devlin RB. TLR-2 is involved in airway epithelial cell response to air pollution particles. Toxicol Appl Pharmacol (2005) 203(1):45–52. doi: 10.1016/j.taap.2004.07.007 15694463

[35] Kitaura H Kimura K Ishida M Kohara H Yoshimatsu M Takano-Yamamoto T. Immunological reaction in TNF-α-mediated osteoclast formation and bone resorption *in vitro* and *in vivo* . Clin Dev Immunol (2013) 2013:181849. doi: 10.1155/2013/181849 23762085 PMC3676982

[36] Ranzani OT Milà C Kulkarni B Kinra S Tonne C. Association of ambient and household air pollution with bone mineral content among adults in peri-urban south India. JAMA Network Open (2020) 3(1):e1918504. doi: 10.1001/jamanetworkopen.2019.18504 31899531 PMC6991311

[37] Yeni YN Kim DG Divine GW Johnson EM Cody DD. Human cancellous bone from T12-L1 vertebrae has unique microstructural and trabecular shear stress properties. Bone (2009) 44(1):130–6. doi: 10.1016/j.bone.2008.09.002 PMC2667817 18848654

[38] Cauley JA Chalhoub D Kassem AM Fuleihan Gel H. Geographic and ethnic disparities in osteoporotic fractures. Nat Rev Endocrinol (2014) 10(6):338–51. doi: 10.1038/nrendo.2014.51 24751883

[39] Zeng Q Li N Wang Q Feng J Sun D Zhang Q. The prevalence of osteoporosis in China, a nationwide, multicenter DXA survey. J Bone mineral Res Off J Am Soc Bone Mineral Res (2019) 34(10):1789–97. doi: 10.1002/jbmr.3757 31067339

[40] Shen L Zhou S Glowacki J. Effects of age and gender on WNT gene expression in human bone marrow stromal cells. J Cell Biochem (2009) 106(2):337–43. doi: 10.1002/jcb.22010 PMC4452949 19115259

[41] Lin YH Wang CF Chiu H Lai BC Tu HP Wu PY. Air pollutants interaction and gender difference on bone mineral density T-score in Taiwanese adults. Int J Environ Res Public Health (2020) 17(24):9165. doi: 10.3390/ijerph17249165 33302461 PMC7764089

[42] Thompson AR Joyce M Stratton K Orwoll ES Carlson HL Carlson NL. Lifetime smoking history and prevalence of osteoporosis and low bone density in U.S. Adults, national health and nutrition examination survey 2005-2010. J women's Health (2023) 32(3):323–31. doi: 10.1089/jwh.2022.0153 36399604

[43] Alvaer K Meyer HE Falch JA Nafstad P Søgaard AJ. Outdoor air pollution and bone mineral density in elderly men - the Oslo Health Study. Osteoporosis Int J established as result cooperation between Eur Foundation Osteoporosis Natl Osteoporosis Foundation USA (2007) 18(12):1669–74. doi: 10.1007/s00198-007-0424-y 17619807

[44] Weng W Bovard D Zanetti F Ehnert S Braun B Uynuk-Ool T. Tobacco heating system has less impact on bone metabolism than cigarette smoke. Food Chem Toxicol an Int J published Br Ind Biol Res Assoc (2023) 173:113637. doi: 10.1016/j.fct.2023.113637 36708864

[45] Gaddini GW Turner RT Grant KA Iwaniec UT. Alcohol: A simple nutrient with complex actions on bone in the adult skeleton. Alcoholism Clin Exp Res (2016) 40(4):657–71. doi: 10.1111/acer.13000 PMC4918769 26971854

[46] Saha H Mukherjee B Bindhani B Ray MR. Changes in RANKL and osteoprotegerin expression after chronic exposure to indoor air pollution as a result of cooking with biomass fuel. J Appl Toxicol JAT (2016) 36(7):969–76. doi: 10.1002/jat.3275 26691826

[47] Prada D Zhong J Colicino E Zanobetti A Schwartz J Dagincourt N. Association of air particulate pollution with bone loss over time and bone fracture risk: analysis of data from two independent studies. Lancet Planetary Health (2017) 1(8):e337–e47. doi: 10.1016/S2542-5196(17)30136-5 PMC5841468 29527596

[48] Wu CH Chang YF Chen CH Lewiecki EM Wüster C Reid I. Consensus statement on the use of bone turnover markers for short-term monitoring of osteoporosis treatment in the asia-pacific region. J Clin densitometry Off J Int Soc Clin Densitometry (2021) 24(1):3–13. doi: 10.1016/j.jocd.2019.03.004 31010789

[49] Brown JP Don-Wauchope A Douville P Albert C Vasikaran SD. Current use of bone turnover markers in the management of osteoporosis. Clin Biochem (2022) 109-110:1–10. doi: 10.1016/j.clinbiochem.2022.09.002 36096182

[50] Watts NB Camacho PM Lewiecki EM Petak SM. American association of clinical endocrinologists/american college of endocrinology clinical practice guidelines for the diagnosis and treatment of postmenopausal osteoporosis-2020 update. Endocrine Pract Off J Am Coll Endocrinol Am Assoc Clin Endocrinologists (2021) 27(4):379–80. doi: 10.1016/j.eprac.2021.02.001 33577971

[51] Choi R Lee SG Lee EH. Intra-individual changes in total procollagen-type 1 N-terminal propeptide in a korean adult population. Diagnostics (Basel Switzerland) (2022) 12(10):2399. doi: 10.3390/diagnostics12102399 36292087 PMC9601271

[52] Eastell R Pigott T Gossiel F Naylor KE Walsh JS Peel NFA. DIAGNOSIS OF ENDOCRINE DISEASE: Bone turnover markers: are they clinically useful? Eur J Endocrinol (2018) 178(1):R19–r31. doi: 10.1530/EJE-17-0585 29046326

[53] Liu C Fuertes E Flexeder C Hofbauer LC Berdel D Hoffmann B. Associations between ambient air pollution and bone turnover markers in 10-year old children: results from the GINIplus and LISAplus studies. Int J hygiene Environ Health (2015) 218(1):58–65. doi: 10.1016/j.ijheh.2014.07.006 25153026

[54] Feizabad E Hossein-Nezhad A Maghbooli Z Ramezani M Hashemian R Moattari S. Impact of air pollution on vitamin D deficiency and bone health in adolescents. Arch Osteoporosis (2017) 12(1):34. doi: 10.1007/s11657-017-0323-6 28378273

[55] Kheirouri S Alizadeh M Abad RMS Barkabi-Zanjani S Mesgari-Abbasi M. Effects of sulfur dioxide, ozone, and ambient air pollution on bone metabolism related biochemical parameters in a rat model. Environ Analysis Health Toxicol (2020) 35(4):e2020023–0. doi: 10.5620/eaht.2020023 PMC7829409 33434423

